# Local and systemic changes in expression of resistance genes, *nb-lrr* genes and their putative microRNAs in Norway spruce after wounding and inoculation with the pathogen *Ceratocystis polonica*

**DOI:** 10.1186/1471-2229-12-105

**Published:** 2012-07-09

**Authors:** Carl Gunnar Fossdal, Nadeem Yaqoob, Paal Krokene, Harald Kvaalen, Halvor Solheim, Igor A Yakovlev

**Affiliations:** 1 Norwegian Forest and Landscape Institute, Høgskoleveien 8, As, NO-1431, Norway; 2 Department of Ecology and Natural Resource Management, Norwegian University of Life Sciences, Høgskoleveien 12, As, NO-1432, Norway

**Keywords:** qRT–PCR, Resistance, *Ceratocystis polonica*, Necrotroph, *Picea abies*, MicroRNA

## Abstract

**Background:**

NB-LRR resistance proteins are involved in recognizing pathogens and other exogenous stressors in plants. Resistance proteins are the first step in induced defence responses and a better understanding of their regulation is important to understand the mechanisms of plant defence. Much of the post-transcriptional regulation in plants is controlled by microRNAs (miRNA). We examined the expression of five Norway spruce miRNA that may regulate NB-LRR related transcripts in secondary phloem (bark) of resistant Norway spruce after wounding and inoculation with the necrotrophic blue stain fungus *Ceratocystis polonica*.

**Results:**

The plants of this clone recovered from both the pathogen inoculations and wounding alone. We found local and systemic induction of the resistance marker genes *PaChi4, PaPAL* and *PaPX3* indicative of an effective induced host defence response. There were minor local and systemic changes in the expression of five miRNAs and 21 NB-LRRs between healthy and treated plants. Only five putative NB-LRRs (*PaLRR1, PaLRR3, PaLRR14, PaLRR15* and *PaLRR16*) showed significant increases greater than two-fold as a local response to *C. polonica*. Of all NB-LRRs only *PaLRR3*, the most highly differentially regulated NB-LRR, showed a significant increase also due to wounding. The five miRNAs showed indications of an initial local and systemic down-regulation at day 1, followed by a later increase up to and beyond the constitutive levels at day 6. However, the initial down-regulation was significant only for *miR3693* and *miR3705*.

**Conclusions:**

Overall, local and systemic expression changes were evident only for the established resistance marker genes and *PaLRR3*. The minor expression changes observed both for the followed miRNAs and their predicted NB-LRR targets suggest that the expression of most NB-LRR genes are maintained close to their constitutive levels in stressed and healthy Norway spruce plants.

## Background

The ability of individual plants to recognize pathogens depends on the plant’s complement of resistance genes [1,2]. Plant immunity is considered to rely on two major levels of resistance [3] and references therein]. The first level is induced by the recognition of conserved microbial molecules called pathogen associated molecular patterns (PAMPs) by cell surface located pattern recognition receptors (PRRs) that induce PAMP triggered immunity (PTI). PRRs are transmembrane proteins with extracellular LRR domain. The most classical example of PTI is the *Arabidopsis* resistance gene FLS2 coding for a LRR-kinase that recognize the PAMP in bacterial flagellin [4], that can be considered as a general or non-host type resistance. However, host-adapted pathogens evolve effectors (AVRs) that counteract PTI [3]. Host plants may then utilize the second level of resistance known as effector triggered immunity (ETI), to recognize and counteract the effectors from adapted pathogens. ETI is mediated by resistance proteins that directly or indirectly perceive pathogen effectors (AVR) proteins. Most ETI receptors are resistance proteins with nucleotide-binding site and leucine-rich repeat (NB-LRR) domain. In flax, TIR-NB-LRRs rust resistance proteins are involved in recognizing effectors from pathogenic rust triggering ETI in a gene-for-gene specific manner [3,5].

The most prevalent class of disease resistance proteins involved in pathogen recognition are NB-LRRs [1,6,7]. NB-LRR genes encode for a variable N-terminal domain of approximately 200 amino acids, and a variable C-terminal tandem array of short LRR motifs. Dependent on the motif within their N-terminus NB-LRRs are categorized into several subgroups, including the Toll/interleukin-1 receptor (TIR) group, the coiled-coil (CC) group, and the leucine zipper (LZ) group. Additionally, the LRR kinase family contains extra-cytoplasmic LRRs fused with a cytoplasmic serine-threonine kinase (KIN) domain [1,5,8]. The LRR domain of NB-LRRs interacts directly or indirectly with the products called effectors of pathogen AVR genes and is hence thought to function primarily in pathogen recognition [1,3,5,8,9]. In angiosperms NB-LRRs have been shown to have a direct role in plant resistance by turning on downstream defence genes [1,4,10]. Receptor oligomerization is likely an important step in the activation of ETI resistance signaling by NB-LRRs [3]. However, a clear model of the signaling events that link NB-LRR activation to the downstream immune responses remains elusive, but direct or indirect interaction with transcriptional regulators such as WRKYs are likely involved [10].

The extensive recent R-gene duplications that have taken place in the perennial angiosperms grapevine and poplar compared with annuals such as rice and *Arabidopsis*, suggests that NB-LRR-encoding gene expansion is a mechanism to compensate for the longer generation time in woody perennials [11]. Kohler and co-workers [12] detected over 400 NB-LRRs in the black cottonwood *Populus trichocarpa* genome, and this is at least twice the number found in *Arabidopsis*. NB-LRRs are also found in gymnosperms such as pine, where they have been tightly linked to resistance to e.g. white pine blister rust *Cronartium ribicola*[13,14]. Most work on NB-LRRs in conifers has been done on western white pine *Pinus monticola*, where Liu and Ekramoddoullah found a large number of TIR-NB-LRRs as well as 61 transcripts of the CC-NB-LRR subfamily [13,14]. The NB-LRRs thus seem to be multifarious in long lived plants such as trees.

The necrotrophic blue stain fungus *Ceratocystis polonica* (Siem.) C. Moreau, is a virulent associate of the Eurasian spruce bark beetle *Ips typographus* L. [15]. The pathogen *C.* polonica is able to kill the host Norway spruce, and is considered the causal agent of tree death rather than the beetles that vector it during bark beetle mass outbreaks. Norway spruce trees pre-treated with mechanical wounding are much more resistant than untreated trees to subsequent challenge inoculation with this necrotrophic blue stain fungus [15]. Similarly, acquired resistance has been observed in response to previous fungal inoculations, resulting in smaller lesion length and less damage upon a second challenge with massive *C. polonica* inoculation [16]. The interaction between spruce and *C. polonica* is likely very ancient and this pathogen is thus likely highly co-evolved and well adapted to its host. This raises the possibility that there is a highly refined suite of NB-LRR type host receptors and ETI related responses involved. However, it is not known to what extent NB-LRRs are involved in pathogen recognition and wound associated responses in spruce.

Recognition of the pathogen and other stresses induce signalling pathways that again cause expression of the downstream genes such as the ones coding for pathogenesis related proteins and enzymes in the phenylpropanoid pathway. Genes regulating lignin metabolism are important for angiosperms resistance to fungal pathogens [17] as plants seem to use increased cell wall lignification to combat invading pathogens. Lignin related peroxidases that are typically up-regulated in both angiosperm and gymnosperm plants after pathogen invasion cause increased lignification and suberization of the host tissues, as well as production of reactive oxygen species [18-20]. The lignin related peroxidase *PaPX3* has a general stress-induced function and is upregulated in Norway spruce bark in response to infection by the root rot fungus *Heterobasidion parviporum* and wounding [20]. Phenylalanine ammonia-lyase (*PaPAL*) also has a key role in lignin and phenylpropanoid formation in gymnosperms such as spruce and in angiosperm plants [20-22]. In Norway spruce a host of defense related proteins are upregulated in response to pathogen attack and other stressors, and the chitinase *PaChi4* has proven to be a particularly useful marker for local and systemic defence response in both Norway spruce and Sitka spruce [23-25].

MicroRNAs (miRNAs) that are involved in plant innate immunity triggered by pathogen associated molecular patterns (PAMPs) have been identified in *Arabidopsis*[26]. miRNAs are post-transcriptional regulators that bind to complementary sequences on target messenger RNA transcripts, usually resulting in translational repression and gene silencing. These regulators are short ribonucleic acid molecules that are on average only 21 nucleotides long in pine and Norway spruce [27,28]. In loblolly pine *Pinus taeda* miRNAs have been associated with the fusiform rust gall disease [27]. The importance of miRNA in spruce defence against pathogens is unknown, but miRNAs targeting stress related genes were overrepresented in small RNA libraries from Norway spruce. Among these were five miRNAs putatively targeting NB-LRR transcripts [28].

NB-LRRs have been identified in spruce and there is now a wealth of available transcript sequences that makes it possible to identify NB-LRRs *in silico*, but the exact number of these genes in conifers such as Norway spruce and other gymnosperms is unknown. Here we follow the differential expression of 21 NB-LRR related genes and five putative miRNAs targeting these genes, as well as the defence response markers *PaChi4, PaPX3* and *PaPAL* in Norway spruce over a spatial and temporal gradient. Expression profiling of these key genes will clarify whether transcriptional regulation of NB-LRRs or miRNAs is a key feature in the local and systemic resistance responses in Norway spruce, and reveal if there is a differential host response to wounding and pathogen infection.

## Results

### Induction of host defence genes

**Figure 1:**
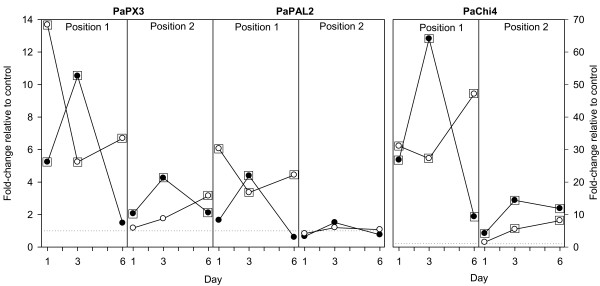
**Expression profiles of three induced defence marker genes in the bark of Norway spruce 1–6 days after inoculation with the necrotroph*Ceratocystis polonica*(white dots) or wounding (black dots).** Data points surrounded by a square are significant different from the control [p&0.05]. Trees were sampled at the treatment site (Position1) and 2–3 cm above the treatment site (Position 2). Gene expression is given as fold change relative to intact control trees at each time point. Dotted horizontal lines indicate equal expression in treatments and controls. n=2 trees.

Transcript levels of the class III peroxidase *PaPX3* and the class IV chitinase *PaChi4* increased significantly both locally and systemically in the bark following *C. polonica* inoculation and wounding (Figure 1). The local host response to inoculation with *C. polonica* was faster than to wounding alone. The systemic increase was lower and delayed compared to the local response, and the systemic response to wounding was stronger than to *C. polonica*. The phenylalanine ammonia-lyase *PaPAL* increased locally in response to inoculation and wounding, but did not change significantly systemically. The host response to wounding alone peaked at day 3 for all three transcripts both locally and systemically and then dropped towards constitutive levels, while transcript levels tended to stay more elevated in response to *C. polonica* throughout the 6day experimental period.

### Organization and phylogeny of NB-LRR genes in spruce

Broad searches for genes in the NB-LRR family in the NCBI and DFCI databases revealed more than 200 different *Picea* entries containing the NB-LRR motif (Additional file 1, 2 and 3:). The entries were divergent in both nucleotide and amino acid sequence and could be divided into six clusters (RI family NB-LRRs, NB-LRRs, CC-NB-LRRs, TIR-P-loop-NB-LRRs, NB-LRR receptor-like kinases, Plant intracellular Ras-group-related NB-LRRs). The clusters were primarily based on presence of TIR or CC domains and additional internal motifs like the protein kinase domain, the NB-ARC domain, F-box and others. We found no BED-finger-NB-LRRs. Clustering analyses of the selected NB-LRR gene models based on similarities in their translated amino acid sequences are presented in Additional file 3.

Five putative NB-LRR-targeting miRNAs were identified (*PamiR950, PamiR9501, PamiR3693, PamiR3697* and *PamiR3705*) [28], and based on sequence similarity their putative targets were determined to be *PaLRR25, PaLRR26, PaLRR27, PaLRR28* and *PaLRR29*, respectively (Additional file 2: Table s 1.2 and Table s 1.2). Of these five NB-LRRs, *PaLRR27* is predicted to be a CC-NB-LRR, while the remaining four are predicted to be TIR-NB-LRRs (Additional file 3: Supplement 3). Twenty-one NB-LRRs and the five miRNAs were followed by real-time RT-PCR (qRT-PCR) and the primer pairs used invariably gave a single PCR product with a corresponding single melting point per target (Additional files 1, 2 and 4).

### Differential expression of putative NB-LRRs in response to inoculation and wounding

**Figure 2:**
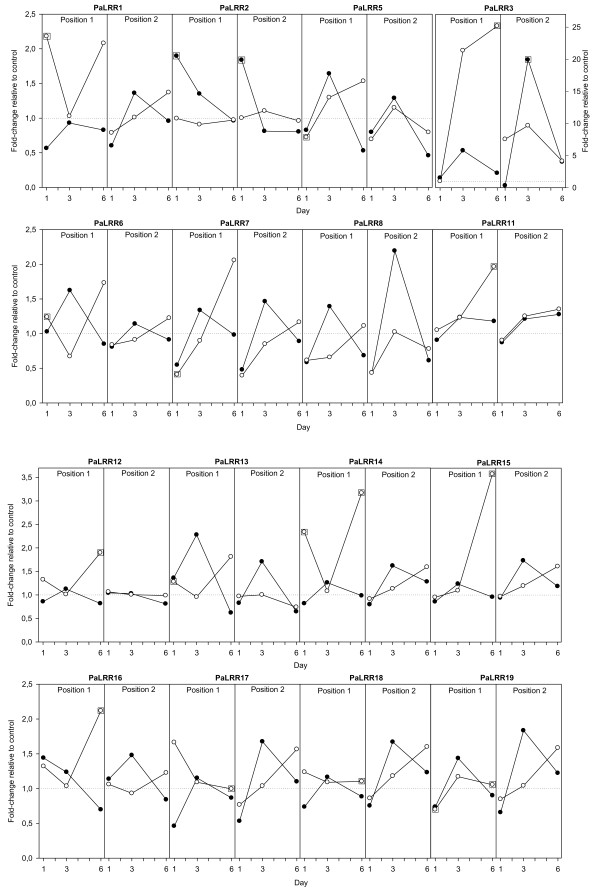
**Expression profiles of 16 NB-LRRs (PaLRRs) in the bark of Norway spruce 1–6days after inoculation with the necrotroph*Ceratocystis polonica*(white dots) or wounding (black dots).** Data points surrounded by a square are significant different from the control [p&0.05]. Trees were sampled at the treatment site (Position1) and 2–3 cm above the treatment site (Position 2). Gene expression is given as fold change relative to intact control trees at each time point. Dotted horizontal lines indicate equal expression in treatments and controls. n=2 trees.

**Figure 3:**
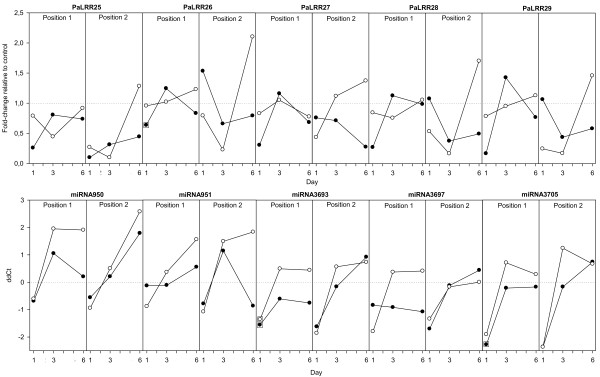
**Expression profiles of five miRNAs and their putative NB-LRR (PaLRR) targets in the bark of Norway spruce 1–6 days after inoculation with the necrotroph*Ceratocystis polonica*(white dots) or wounding (black dots).** Data points surrounded by a square are significant different from the control [p&0.05]. Trees were sampled at the treatment site (Position1) and 2–3 cm above the treatment site (Position 2). For LRRs expression levels are given as fold change relative to intact control trees at each time point. For miRNAs expression is given as relative PCR cycle differences (ddCt). Dotted horizontal lines indicate equal expression in treatments and controls. n=2 trees.

Most of the quantified NB-LRR transcripts remained very close to constitutive levels or showed only minor transcript changes across treatments and time points (*PaLRRs 2, 5, 6, 7, 8, 11, 12, 13, 17, 18, 19, 25, 26, 27, 28* and *29*; Figures 2 and 3). Only five NB-LRRs (*PaLRR1 PaLRR3*, *PaLRR14, PaLRR15* and *PaLRR16*) showed more than two-fold significant changes between control and treatments (Figure 2). The CC-NB-LRR-related *PaLRR3* had the singularly largest increases in expression, with up to 20-fold systemic increase in response to wounding alone and up to 25-fold local increase due to *C. polonica.*

### Differential expression of miRNAs in response to inoculation and wounding

For the miRNAs changes in transcript abundance are given as relative differences in the number of PCR cycles (ddCt), so here a value of 1 equals 2^1^, i.e. a two-fold increase. This approach differs slightly from the absolute quantitative values given for the NB-LRR-like transcripts. The five studied miRNAs showed a largely similar trend in expression in response to both wounding and fungal inoculation (Figure 3). The rapid down-regulation of miRNAs in both wounded and fungus inoculated samples at day 1 was significant for *PamiR3693* and *PamiR3705*. Due to variation between biological replicates the later increase observed for some miRNAs on day 3 and 6 was not significant. The five putative miRNA targets *PaLRRs 25* to *29* did not show expression patterns that co-varied in concert with their corresponding miRNAs, i.e. there was no consistent increase in putative target PaLRRs when miRNAs levels decreased on day 1.

## Discussion

This work is the first attempt to survey the diversity of NB-LRRs in spruce and to follow the expression pattern of miRNAs putatively targeting resistance genes in plants. Expression of host defence marker genes was induced both locally and systemically in response to both wounding alone and inoculation with the pathogen *C. polonica.* The plants seemed to recover quickly from the treatments, confirming this clone’s high level of resistance. Most of the NB-LRRs we studied remained close to their constitutive levels in both healthy, wounded and pathogen inoculated Norway spruce. However, *PaLRR3* (a CC-NB-LRR type resistance gene) showed extensive local and systemic increase in response to wounding and inoculation. We found a rapid drop in miRNAs putatively targeting NB-LRRs, but the change was significant only for two miRNAs and there was no corresponding change in their putative NB-LRR targets.

Surprisingly little is known about how NB-LRRs and related genes are regulated after pathogen attack and other stresses in long lived plants such as trees, and this even includes the well-studied *Populus* tree species [6]. Previous studies have established that Norway spruce trees resistant to necrotrophic pathogens have rapid induction of defence related genes (e.g. peroxidases, class IV chitinase and others) [24,29], and also appear to have more efficient systemic defence signalling than susceptible genotypes. In the current work with a relatively resistant Norway spruce clone we found indications that not only downstream genes such as *PaPalPaPX3* and *PaChi4,* but also some NB-LRRs-like transcripts and two putatively regulatory miRNAs, are differentially regulated locally and systemically following fungal inoculation and wounding. However, this is overshadowed by the main finding that most NB-LRRs appear to be under very strict transcriptional control and are maintained at low and stable constitutive levels in both healthy and stressed plants. The maintenance of stable low levels of most NB-LRRS might be related to the cost of preserving these large proteins and associated signalling pathways, and the fact that even low constitutive levels may be sufficient to respond effectively to foreign invaders.

NB-LRR genes appear to be abundant in the conifer Norway spruce, but since we do not have the genome sequence of Norway spruce we cannot at this time determine the true number of resistance genes present. We found more than 200 LRR-containing resistance-like genes in the available spruce databases, and this is similar to other plant species. More than 160 NB-LRR-encoding genes have been identified in the Col-0 *Arabidopsis* genome [30], 250 full-length NB-LRR genes and 560 NBS sequences are known from rice [31], and so far more than 400 resistance-like genes have been identified in *Populus*[12]. Among conifer trees NB-LRR genes have been most studied in pines. Western white pine shows genetic variation in disease resistance to white pine blister rust and in their TIR-NB-LRR and CC-NB-LRR genes [13,14]. Liu and Ekramoddoullah [13] proposed that conifers have a large and diverse NB-LRR gene family and that conifer resistance genes share a common origin with R genes from angiosperms. NB-LRRs have been reported in several conifers, including loblolly pine *Pinus taeda* (L.) [32], Japanese cedar *Cryptomeria japonica*[33], Norway spruce [29], sugar pine *Pinus lambertiana* and western white pine [34,35]. There is now large numbers of transcripts available from spruces and other conifers. We found 259 LRR matching sequences at the DFCI Spruce Gene Index (Sgi, Release 5.0, dt. 30.03.2011) ( http://compbio.dfci.harvard.edu/cgi-bin/tgi/gimain.pl?gudb=spruce). *In silico* analysis of these confirms that spruce has a reasonably large repertoire of defence and resistance-like genes, and in our search we found all the major subgroups of LRR genes, except the BED-finger LRR protein genes reported from *Populus*[6,36].

NB-LRRs have been linked to resistance in conifers. In white pine the TIR-NB-LRR homologue *PmTNL1* is a resistance gene linked to partial resistance to white pine blister rust *C. ribicola*. The *PmTNL1* transcript is expressed at low basal levels in different tissues. Expression remained unchanged during compatible and incompatible interactions with *C. ribicola* at the early stages post-inoculation, but in later stages of the interaction higher levels were found in symptomatic plants [35]. We saw a similar but less evident upregulation for the RI family NB-LRR *PaLRR15,* as well as for *PaLRR1PaLRR14* and *PaLRR16*, and most notably for the CC-NB-LRR class *PaLRR3*. However, most of the TIR-NB-LRRs and other NB-LRRs that we studied remained at stable levels following wounding or inoculation. This agrees with previous studies showing that resistance genes such as NB-LRRs typically are expressed constitutively at low basal levels and do not increase notably after pathogen challenge [35-37]. Similar results were found in a comprehensive study of NB-LRRs in *Populus*. Kohler and coworkers, examining the diversity of NB-LRR genes in the *Populus trichocarpa* genome, detected only 34 of 400 known NB-LRR homologues from rust-infected and non-infected leaves using a whole-genome oligoarray, and none of the 34 NB-LRRs showed an altered expression two days post-inoculation [12]. Our results from Norway spruce thus support the hypothesis that NB-LRRs generally are kept close to their constitutive levels. However, it cannot be ruled out that at least some R-like genes are regulated at the transcriptional or posttranscriptional level within hours after an attack, and it is also conceivable that the constitutive level of individual NB-LRRs may be heightened in primed plants [38].

Our transcriptional data are not sufficient to establish a clear link between the expression of the miRNAs *PamiR950*, *PamiR951*, *PamiR3693*, *PamiR3697* and *PamiR3705*, their NB-LRR candidates *PaLRR25* to *29*, and the downstream defence induction markers in Norway spruce. However, it was expected that the rapid and statistically significant down-regulation of *PamiR3697* and *PamiR3705* would be mirrored by greater expression of their putative NB-LRR targets. There could be several explanations for the lack of a clear correspondence between the expression of miRNAs and their predicted NB-LRR target sequences; (1) the change in miRNA levels detected here may simply be too small to have a significant impact on NB-LRR targets, (2) the miRNAs may act preferentially on other NB-LRR paralogues than the ones identified here, (3) the putative NB-LRR targets could be also the origin of the miRNAs, complicating matters even further, (4) the expression of the NB-LRR target genes may be under tight control of positive and negative regulators that bind their promoters, thus overriding the effect of miRNAs, or less likely (5) that these miRNAs might inhibit the translation of NB-LRRs and not cause breakdown of the RNA transcripts. The most likely explanation is probably that the small changes we detected in miRNA levels work in concert with regulatory changes by transcription factors at the promotor of these genes in order to maintain stable NB-LRR transcript levels. It is also possible that we should have looked at changes in miRNAs and their NB-LRR targets at a much earlier time point after treatments than day 1 to 6, and preferentially have focused only on the host cells in the immediate vicinity of the inoculation/wounding site. However, our combined results do not rule out that miRNA might be involved in local and systemic defence response in Norway spruce, but show that a much larger set of small RNAs (extending to other targets than NB-LRRs) must be studied in combination with transcriptome and degradome studies of all targets by way of latest generation sequencing methods.

Specific cell types in a tissue may have specialized roles in pathogen recognition and systemic defense signaling that are not resolved when collecting samples from large sections of tissues such as bark. The bark of a conifer contains specialized tissues (phloem, cortex and periderm) with specialized cells that are organized in a regular pattern [39], such as the rays running through the bark into the wood that can play specialized roles in defense. Thus, the defense responses might be highly cell type specific or expressed in a subtle gradient across the diseased tissue stressing the need for using high resolution methods such as in-situ hybridization and Laser micro dissection (LMD) approaches. LMD of conifer stem tissues for the isolation individual cell types for transcript analysis, enzyme activity and metabolites has already been perform on individual cell types (resin ducts and cambial zone) in the bark of white spruce [39]. To better establish the role of resistance genes, miRNAs and signalling in Norway spruce, such LMD based experiments are now needed in order to get better insight into the recognition of pathogens and their effectors at the single cell type level.

## Conclusions

Two conclusions can be drawn from our data. First, most of the NB-LRRs in the gymnosperm Norway spruce are maintained at low constitutive levels, suggesting that basal levels of these gene products are sufficient to serve their putative resistance gene function as receptors (or translocators) of external challenges such as a pathogen. Thus, the NB-LRRs’ role in resistance may be to be constitutively present to detect pathogen effectors (directly or indirectly), and not to be differentially regulated following a challenge [6,8,11,26,35-37].

Secondly, in this first study of putative NB-LRR targeting miRNAs we observed relatively minor changes in expression of miRNAs and their predicted NB-LRR targets. This does not necessarily rule out a role for miRNAs in resistance, but suggests that they may, in concert with other regulators, contribute to the tight constitutive expression of NB-LRRs needed to detect external challenges.

## Methods

### Plant material and sampling

The experimental material consisted of 21 identical 2-year-old saplings generated by somatic embryogenesis from a single Norway spruce clone. This clone (AL15886-B10) was derived from a single seed from a full-sib cross (♀ #2650 × ♂ #2707) and the saplings were produced at the Biri Nursery and Seed Improvement Centre, Norway [40]. AL15886-B10 is relatively resistant to fungal infection, as it contains the fungus within very short necrotic lesions in the bark and recovers within a month after inoculation with the necrotrophs *Heterobasidion parviporum* and *Ceratocystis polonica*[25]. After having formed buds under natural light conditions and overwintering as previously described [41] the genetically identical saplings were exposed to late spring conditions and kept in growth chambers under optimal temperatures (20-22 °C) at ambient day length, with 200-250 μmol m^-2^ s^-1^ light conditions. The plants were inoculated and wounded at the time when the leader shoot had extended (actively growing) and well prior to any new bud formation. The plants were well-watered and provided with sufficient fertilizers throughout the experiment [25].

Saplings were inoculated with *C. polonica* (isolate no.NISK 93–208:115) (Cp) or wounded (W) on the stem. Plants were inoculated 5 cm above the ground by cutting a ~5 mm wide bark flap using a scalpel. A small amount of malt agar (1% malt and 1.5% agar) containing actively growing mycelium was placed underneath the bark flap, the flap was pressed firmly against the stem, and the wound was sealed with parafilm. Wounding was done in the same way, but using malt agar without fungus. Unwounded control plants (C) were also wrapped with parafilm at the corresponding position of the stem. Local and more distal samples of bark (1 cm long) were harvested 1, 3 and 6 days after treatment from at least two ramets per treatment and time point. Samples were quick-frozen in liquid nitrogen and stored at −80°C until analysis. To distinguish between local and systemic gene expression local and distal samples were analysed separately. The local sample (W1/Cp1) included the inoculation/wounding site and extended ca 0.5 cm above the upper margin of the wound. The distal sample (W2/Cp2) included the entire bark region 2–3 cm above the inoculation/wounding site. The unwounded controls were sampled at the corresponding positions.

### NB-LRR gene annotation, peptide structure and phylogenetic analyses

Initial searches for NB-LRR genes were performed using the NB-LRR family conserved catalytic domain to probe the *Picea* (taxid: 3328) sequences at the National Centre for Biotechnology Information (NCBI) database using BLASTP algorithms. The conserved domain was derived from a range of NB-LRR gene sequences available from NCBI GenBank ( http://www.ncbi.nlm.nih.gov/). All the unique sequences we obtained were verified using the NCBI blast server with BLASTP searches against the NCBI GenBank and the Conserved Domain Database (CDD) [41]. Gene models containing NB-LRR motifs were chosen for further examination. In addition, we searched the DFCI Picea Gene Index (release 5.0, March 30, 2011 http://compbio.dfci.harvard.edu/cgi-bin/tgi/gireport.pl?gudb=spruce) using the keyword “LRR” to select a list of unigenes temporarily annotated as NB-LRR motif-containing genes.

We conducted phylogenetic and molecular evolutionary analyses for translated amino acid sequences of the selected NB-LRR gene models using the MEGA4 software [42]. The evolutionary history was inferred using the Neighbour-Joining method. The bootstrap consensus tree inferred from 500 replicates is taken to represent the evolutionary history of the taxa analysed. Branches corresponding to partitions reproduced in less than 50% of the bootstrap replicates were collapsed. The phylogenetic tree was linearized assuming equal evolutionary rates in all lineages. The tree is drawn to scale, with branch lengths in the same units as the evolutionary distances used to infer the phylogenetic tree. The evolutionary distances were computed using the Poisson correction method and are expressed as the number of amino acid substitutions per site. Positions containing alignment gaps and missing data were eliminated only in pairwise sequence comparisons (Pairwise deletion option).

### Selection of miRNAs putatively targeting NB-LRRs

We identified five miRNAs putatively targeting the NB-LRR transcripts *PaLRR25PaLRR26PaLRR27PaLRR28* and *PaLRR29* (table s1.2). These miRNAs had all the *in silico* characteristics of microRNA identified in an earlier study of miRNAs involved in epigenetic regulation and climatic adaptation in Norway spruce [28]. The GenBank accession numbers for the miRNAs putatively targeting NB-LRRs are given in Additional file 2; Table s2, and the accession numbers for the NB-LRR sequences are given in Additional file 1: Table s1.1, Additional file 2: Table s1.2 and Additional file 3: Supplement 3.

### Total RNA extractions and cDNA synthesis

Bark samples (100–150 mg) were ground first in liquid nitrogen using a mortar and a pestle and then transferred to 2 ml eppendorf tubes for fine grinding in a Retsch 300 Mill (Retsch Gmbh, Haan, Germany) for 1.5 min. The equipment and tissue samples were kept chilled with N_2_ throughout the grinding process. Total RNA (including the small RNA fraction) was extracted from 100 mg of the pulverized samples using the Master Pure™ RNA Purification Kit (Epicentre, Madison, WI, USA, #MCR85102) following manufacturer recommendations and stored at −80°C until further use. RNA was quantified using a micro-volume spectrophotometer (Nano Drop 2000, Thermo Scientific, Wilmington, DE, USA).

### cDNA synthesis and real-time RT-PCR of target transcripts

Gene specific primers were designed using Primer3 [43] with the criteria of melting temperature 70°C and product size&120 bp. All the studied NB-LRR-like homologs and their primer sequences are listed in table s1.1. The specificity of all primers was tested by running the PCR product on a 2% agarose gel after RT-PCR amplification. Only primers producing clear single bands were used in the RT-PCR experiment (Additional file 4: Figure s4). The primers used for quantification of *PaChi4, PaPAL* and *PaPX3* are previously verified [23]. Total RNA was reverse transcribed (1 μg per reaction) using the TaqMan Reverse Transcription kit (Applied Biosystems, Carlsbad, CA, USA, # 8080234) in 50 μl reaction volume and diluted to 200 μl. PCR amplification was performed in a 25 μl reaction volume, using 2 μl of diluted cDNA solution as template, 12.5 μl of 1x SYBR Green master mix and 200 nM of each primer. RT-PCRs were performed using the 7500 Fast Real-time PCR System (Applied Biosystems, Carlsbad CA, USA) with standard cycling parameters. All reactions were run in duplicate and a no-template control was run for each primer pair. For data analysis, the arithmetic mean of two biological replicates was calculated. Target gene expression was normalized to the transcript level of actin (*PaAct*). In our previous work the *PaAct* transcript has proved to be the best endogenous reference for RT-PCR in Norway spruce as it gives the better stability index when compared to alphaTubulin (*PaTub*), Polyubiquitin (*PaUbq*) and Glyceraldehyde-3-phosphate dehydrogenase (*PaGAPDH*) [44]. To further ensure that *PaAc*t was a stable reference we compared the stability of *PaAct* expression levels in all the day one samples against *PaTub* and the translation initiation factor (*PaTif*) and found that using *PaAct* alone as endogenous reference for RT-PCR was equally significant as using the combination of the three references *PaActPaTub* and *PaTif* (data not shown).

Absolute quantification was performed using the 7500-system SDS software and data were further processed in MS Excel.

### Quantification of miRNAs

Transcript abundance of the selected miRNAs was determined with relative real-time RT-PCR as described by Yakovlev et al. (2010). cDNAs were synthesized from 600ng of small RNA using the NCode miRNA First-Strand cDNA Synthesis Kit (MIRC-50; Invitrogen) following the manufacturer’s recommendations. Real-time RT-PCR amplification was performed using NCode SYBR GreenER miRNA qRT-PCR Kit (MIRQER-100; Invitrogen) in a 25μl reaction volume, using 2μl of a diluted cDNA solution as template and 200 nM of each primer. Reactions were conducted on the 7500 Real-time PCR System (Applied Biosystems, Foster City, CA, USA) using the Invitrogen recommended cycling conditions. After PCR, dissociation curves were made to verify the specificity of the amplification. There were two technical and two biological replicates for each sample. All miRNA expression levels were normalized to the geometric mean of three selected ribosomal and transfer RNA genes. Forward primers were designed based on mature miRNA sequences. If Tm of the mature miRNA was&60°C, it was adjusted by adding Gs and Cs to the 5-end and/or the 3-end of the miRNA sequence. To verify the specificity of the miRNA amplification we analysed several PCR samples for each miRNAs on 2% agarose gels with ethidium bromide (EtBr) visualization of bands. Reverse primers was supplied with the NCode miRNA First-Strand cDNA Synthesis Kit (MIRC-50; Invitrogen). The miRNA primers used are listed in Additional file 2: Table s1.2 and Additional file 2 Table s2.

The relative ddCT quantification method based on the critical cycle threshold values was used for the miRNAs, using the 7500-system SDS software. Data were further processed in MS Excel.

### Statistical analysis

Relative transcript amounts were calculated from raw data obtained from real-time PCR in the form of critical threshold cycle values (dCt) for the miRNA data as described in [25]. For the NB-LRRs and resistance gene markers absolute quantitative values based on standard curves were obtained [23]. Since several qRT-PCR based observations were made on the same plant for all the plants in the dataset, the transformed values were subjected to repeated measures analysis of variance by use of the GLM and Mixed procedures in the SAS Software system (SAS Institute Inc.,Cary, NC, USA). Planned comparisons between treatment means were performed by use of the pdiff option in the lsmeans statement in these procedures.

## Competing interests

The authors declare that they have no competing interests.

## Authors’ contributions

CGF and PK conceived and designed the study, IAY, PK, CGF and NY performed the experiments and collected the data, HK, IAY, CGF, PK and NY performed the data analysis. CGF, PK, HK, IAY, HS and NY drafted the manuscript. All authors read and approved the final manuscript.

## Supplementary Material

Additional file 1 Table s1.1 Selected NB-LRR family genes in Norway spruce analysed by real time qRT-PCR. Click here for file

Additional file 2 **Table s1.2 The additional selected 5 NB-LRR family genes in Norway spruce putatively regulated by specific miRNAs and analysed by real time qRT-PCR.** Table s2. Selected specific miRNAs putatively targeting NB-LRR family genes (identified in Yakovlev et al., 2010). Click here for file

Additional file 3 **Supplement 3. Phylogenetic tree and cluster description of the selected NB-LRR gene models (full ORF amino acid sequences) based in the spruce ESTs available in the NCBI Databases.** The phylogenetic tree was constructed using MEGA4 as detailed in Materials and Methods. Click here for file

Additional file 4 **Figure s4. Gel showing primer test to identified NB-LRRs in Norway spruce using primers designed from *in silico* studies of exiting spruce sequences in sequence databases.** Only primer pairs giving a single band was further used for real-time RT-PCR. Therefore, NB-LRRs 22, 20, 10, 8 and 4 were not studied by qRT-PCR. Click here for file
