# Supplementary material for: Local and systemic changes in expression of resistance genes, nb-lrr genes and their putative microRNAs in Norway spruce after wounding and inoculation with the pathogen Ceratocystis polonica
Source: BMC Plant Biol. 2012 Jul 9;12:105. doi: 10.1186/1471-2229-12-105 (PMC3431983; doi:10.1186/1471-2229-12-105)
Supplement: Additional file 1 — Table s1.1 Selected NB-LRR family genes in Norway spruce analysed by real time qRT-PCR. [file 1471-2229-12-105-S1.pdf]

Supplement 1

Table s1.1 Selected Nb-LRR family genes in Norway spruce analysed by real time qRT-PCR

Clus-ter	Group	Number	Protein ID	Nucleotide source sequence	Gene name	Primers (5′→ 3′), F/ R	Product length, bp
1	1	1	ABK24805	gi_116788244	PaLRR2	TGGCAAGCCGGAAGATGAGG/ CCAGCAGGGTTTCCAGCAACG	120
2	2	3	ABF73316	gi_104642234	PaLRR11	GCACAAGCGTCCTGCCCATT/ ATCCTTGCCCGTTGCCTTGG	101
		4	ABR18304	gi_148910456	PaLRR12	AAAGTGGCCTTGCTTTGCACTGA/ TGCGACAAAGTATTTCATCATGCACCTT	113
	3	19	ACN41041	gi_224286674	PaLRR13	TGTGGGCCAATCCCAAGAGGAA/ CCACAGGGATGTAATGGCAAACC	97
	4	21	ABR17329	gi_148908431	PaLRR17	CATTGGCTTGACCCGATCCTCTC/ GCTTCAAATGCTTACTCCATGGTTCACA	95
3	5	26	ACN40981	gi_224286550	PaLRR9	GGAGTGCTCAGGATCGAAGAAGATGC/ GCTTTGATGGACAACCACGCTCCTA	111
		27	ABR16233	gi_148906150	PaLRR10	GCCTGATGACCTGGGCAAGTTAAGG/ TCGCAAATGGAAGCTGGAAGCA	90
	6	30	ABK24822	gi_116788283	PaLRR3	CACATTGATCTTCAGGGATGCCACA/ CGGCAGCCATTCCAAATCGT	114
		32	ABR18026	gi_148909879	PaLRR18	GCACAACCGTTCTACCTGTGAGAGAG/ CTGGCTGCTCCTCAAATCTTCCA	106
	7	36	ABR18473	gi_148910813	PaLRR6	TGGTGGAGGATCTCCCGCAAG/ TGCAATTGTGGAAACTCGCAAATACAA	115
		37	ABR18096	gi_148910025	PaLRR7	TGCAATGAGTTGATGAAATTGGACTTG/ TGTACCAAATGGTCCTGTCATGCTCTC	111
4	8	39	ACN39861	gi_224284250	PaLRR8	TGCAAGAACTCAATTTCCAAGGTTGC/ TTTCGCAAGAGGATAAATCCAATATTCTCA	102
	9	40	ABR16849	gi_148907432	PaLRR20	TGGTGCAGCTGACAGGACTTCG/ TTCCGACACCCACCGGCATT	118
	10	47	ABR17027	gi_148907802	PaLRR21	TCCTCCGTTGCCAATGCTCA/ GGGCAGACACCTTCACCAAAGGA	104
	11	54	ADE76430	gi_294461742	PaLRR22	TGCCAAAGTCCCTCGGCAAC/ CCCGCAACTCTTCCAGCGATAACA	114

	12	56	ABR17471	gi_148908727	PaLRR23	CGCCCTACGGTTGGAGGCTGA/ CGCAGTGCATTCTCCCGCTTG	93
5	13	57	ABK24857	gi_116788373	PaLRR19	AAACTCTGATCCCGTGAAGGCAAAC/ CAACATTTGGCCGGGTATGGAA	100
		58	ABK24560	gi_116787569	PaLRR24	TCGACTCCGGGAATGCCCTCT/ AGCGTACTTCCATATCCTCGGCCTTG	90
	14	60	ABR18087	gi_148910004	PaLRR1	TTCCAAGGCAGTGAAGCGTTAGCAG/ CCCGAATGGAACCAGAGCCAAG	90
		62	ABK25485	gi_116790053	PaLRR16	TGGGCTTTCCGTGCTCTCCTT/ AGATAGCCAGGTCTCCAGGGTTTGA	120
		63	ADE76117	gi_294461100	PaLRR14	TGCTCTGCTGGATTGCGAGAGTT/ ACTGCACCCACGGGCTTCCA	106
		65	ABR17804	gi_148909411	PaLRR15	CGACAGTAAGTTGTGGGTGTTCTCAAGG/ CAAACTCGGAATGTTGAATGGCTGGT	103
6	15	72	ABR17008	gi_148907764	PaLRR4	GGGATCGGCGCAAGCAACA/ GCTCTTCAGATTCACACATCCGTCCA	100
		73	ABR18194	gi_148910225	PaLRR5	TGCCTGGTGGAGGTGGACAGG/ GCGCCAAAGACGACACGGATT	108
