# Supplementary material for: Local and systemic changes in expression of resistance genes, nb-lrr genes and their putative microRNAs in Norway spruce after wounding and inoculation with the pathogen Ceratocystis polonica
Source: BMC Plant Biol. 2012 Jul 9;12:105. doi: 10.1186/1471-2229-12-105 (PMC3431983; doi:10.1186/1471-2229-12-105)
Supplement: Additional file 2 — Table s1.2 The additional selected 5 NB-LRR family genes in Norway spruce putatively regulated by specific miRNAs and analysed by real time qRT-PCR. Table s2. Selected specific miRNAs putatively targeting NB-LRR family genes (identified in Yakovlev et al., 2010). [file 1471-2229-12-105-S2.pdf]

Table s1.2. Selected NB-LRR family genes in Norway spruce putatively regulated by specific miRNAs and analysed by real time qRT-PCR

Target LRR gene	Nucleotide source sequence	miRNA ID	Primers (5′→3′), F/ R	Product length, bp	miRNA primer sequence (F)
PaLRR25	ES261905	pab-miR950-3p	TCTCCGTCAGGCGGGTGTTT/ TTGAGGAAGGCAGTTTCAAGCAGAG	80	TCTGGGCCCCGGTGGTTTATGA
PaLRR26	DR563813	pab-miR951	TGCAGAGCGTATTGGATCTTGCTTCT/ GGAGTGGGATGTGGACGAGGATG	88	TGTTCTTGACGTCTGGACCACG
PaLRR27	EX326419	pab-MIR3693	GGAGCTGCTCGTAGGTGAGGGATG/ GAAGTTTGCTCCATTTGAGCGCTTGT	97	AGAGGGTGCTCATGAACTGCTC
PaLRR28	EX427311	pab-MIR3697	CCCTCGCATCCTCCTCATCCA/ TGGCTGGCGAGACCTTTCTTTACATC	106	TAGCCCCTGACTTCAACATGAG
PaLRR29	DR499063	pab-MIR3705	CCGTTAAACTTGCCGCCATCCA/ CAGCCACGATATGACCCCGACA	101	GTAAGTGGTTATGATCTGGAC

Table s2. Selected miRNAs putatively targeting LRR family genes in Norway spruce (Their identification and miRNA precursors are shown in Yakovlev et al., 2010)

miRNA ID	Sequence	Length, nt	miRNA containing EST	Target	Target gene function	Score/ E-value
pab-miR950-3p	TCTGGGCCCCGGTGGTTTATGA	22	ES261905	DR543641	gi|321530320|ADW94527.1 putative TIR-NBS-LRR protein [Pinus monticola]	58.5 bits (140)/ 3e-07
pab-miR951	TGTTCTTGACGTCTGGACCACG	22	DR563813	EX417324 (4 mismatches)	gb|AAM28917.1| putative TIR/NBS/LRR disease resistance protein [Pinus taeda]	157(396)/ 6e-37
pab-MIR3693	AGAGGGTGCTCATGAACTGCTC	22	EX326419	DR492284 (2 mismatches)	gb|AAY78890.1| CC-NBS-LRR resistance-like protein [Pinus lambertiana]	311(796)/ 5e-83
pab-MIR3697	TAGCCCCTGACTTCAACATGAG	22	EX427311	EX417859 (3 mismatches)	gb|AAM28917.1| putative TIR/NBS/LRR disease resistance protein [Pinus taeda]	147(372)/ 2e-42
pab-MIR3705	GTAAGTGGTTATGATCTGGAC	21	DR499063	DR491334 (1 mismatch)	gb|AAM28917.1| putative TIR/NBS/LRR disease resistance protein [Pinus taeda]	212(539)/ 3e-53
