# Supplementary material for: Local and systemic changes in expression of resistance genes, nb-lrr genes and their putative microRNAs in Norway spruce after wounding and inoculation with the pathogen Ceratocystis polonica
Source: BMC Plant Biol. 2012 Jul 9;12:105. doi: 10.1186/1471-2229-12-105 (PMC3431983; doi:10.1186/1471-2229-12-105)
Supplement: Additional file 3 — Supplement 3. Phylogenetic tree and cluster description of the selected NB-LRR gene models (full ORF amino acid sequences) based in the spruce ESTs available in the NCBI Databases. The phylogenetic tree was constructed using MEGA4 as detailed in Materials and Methods. [file 1471-2229-12-105-S3.pdf]

Supplement 3. Phylogenetic tree and cluster description of the selected NB-LRR gene models (full ORF amino acid sequences) based in the spruce ESTs available at the NCBI Database. The phylogenetic tree was constructed using MEGA4 as detailed in Materials and Methods

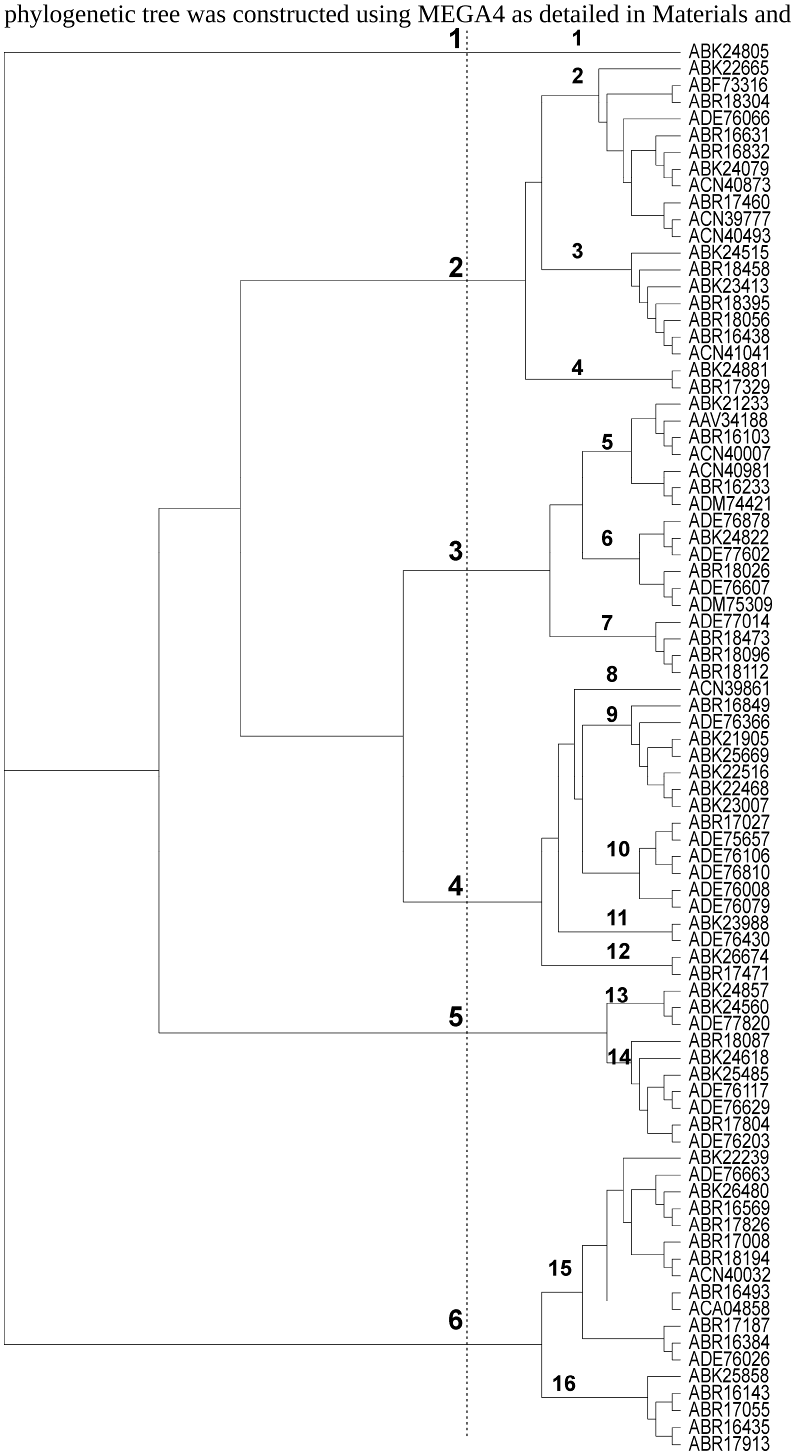

Clus-ter	Group	Description	LRR group
1	1	cd00116: **LRR_RI -** Leucine-rich repeats (LRRs), ribonuclease inhibitor (RI)-like subfamily - 11-residue segments of the Leucine-Rich Repeats (LxxLxLxxN/CxL)	leucine-rich repeat receptor-like protein kinase, RI- like subfamily
2	2	PLN00113[PLN00113], leucine-rich repeat receptor-like protein kinase<br>Pkinase[pfam00069], Protein kinase domain;<br>Including LRR_RI[cd00116], Leucine-rich repeats (LRRs), ribonuclease inhibitor (RI)-like subfamily.	leucine-rich repeat receptor-like protein kinase, RI- like subfamily
	3	PLN00113[PLN00113], leucine-rich repeat receptor-like protein kinase<br>Including LRR_RI[cd00116], Leucine-rich repeats (LRRs), ribonuclease inhibitor (RI)-like subfamily	
	4	PLN00113[PLN00113], leucine-rich repeat receptor-like protein kinase<br>Including LRR_RI[cd00116], Leucine-rich repeats (LRRs), ribonuclease inhibitor (RI)-like subfamily	
3	5	CC-NBS-LRR resistance-like protein, contain NB-ARC[pfam00931], NB-ARC domain and COG4886[COG4886], Leucine-rich repeat (LRR) protein	CC-NBS-LRR resistance-like
	6	PLN00113[PLN00113], leucine-rich repeat receptor-like protein kinase, including COG4886[COG4886], Leucine-rich repeat (LRR) protein	
	7	CC-NBS-LRR resistance-like protein, contain NB-ARC[pfam00931], NB-ARC domain; COG4886[COG4886], Leucine-rich repeat (LRR) protein PLN00113[PLN00113], leucine-rich repeat receptor-like protein kinase	
4	8	TIR-NBS-LRR class	TIR-NBS-LRR class; leucine-rich repeat receptor-like protein kinase
	9	TIR/P-loop/LRR	
	10	PLN00113[PLN00113], leucine-rich repeat receptor-like protein kinase	
	11	PLN00113[PLN00113], leucine-rich repeat receptor-like protein kinase	
	12	COG4886[COG4886], Leucine-rich repeat (LRR) protein; PLN00113[PLN00113], leucine-rich repeat receptor-like protein kinase	
5	13	F-box/LRR-repeat protein; Ran GTPase/Leucine-rich repeats (LRRs), ribonuclease inhibitor(RI)-like subfamily	RanGTPase/Leucine-rich repeats (LRRs), ribonuclease inhibitor(RI)-like
	14	Leucine-rich repeats (LRRs), ribonuclease inhibitor(RI)-like subfamily	
6	15	TIR[pfam01582], TIR domain; The Toll/interleukin-1 receptor (TIR) homology domain; NB-ARC[pfam00931], NB-ARC domain; PLN00113[PLN00113], leucine-rich repeat receptor-like protein kinase	TIR-NBS-LRR class; leucine-rich repeat receptor-like protein kinase
	16	LRRNT_2[pfam08263], Leucine rich repeat N-terminal domain; LRR_RI[cd00116], Leucine-rich repeats (LRRs), ribonuclease inhibitor (RI)-like subfamily; PLN00113[PLN00113], leucine-rich repeat receptor-like protein kinase	
