# Supplementary material for: Local and systemic changes in expression of resistance genes, nb-lrr genes and their putative microRNAs in Norway spruce after wounding and inoculation with the pathogen Ceratocystis polonica
Source: BMC Plant Biol. 2012 Jul 9;12:105. doi: 10.1186/1471-2229-12-105 (PMC3431983; doi:10.1186/1471-2229-12-105)

Supplement 4. Gel showing primer test of the identified NB-LRRs in Norway spruce. Only primer pairs giving a single band was further used for real-time RT-PCR. Therefore, NB-LRRs 22, 20, 10, 8 and 4 were not studied by qRT-PCR. DNA size ladder is represented by the letter L.

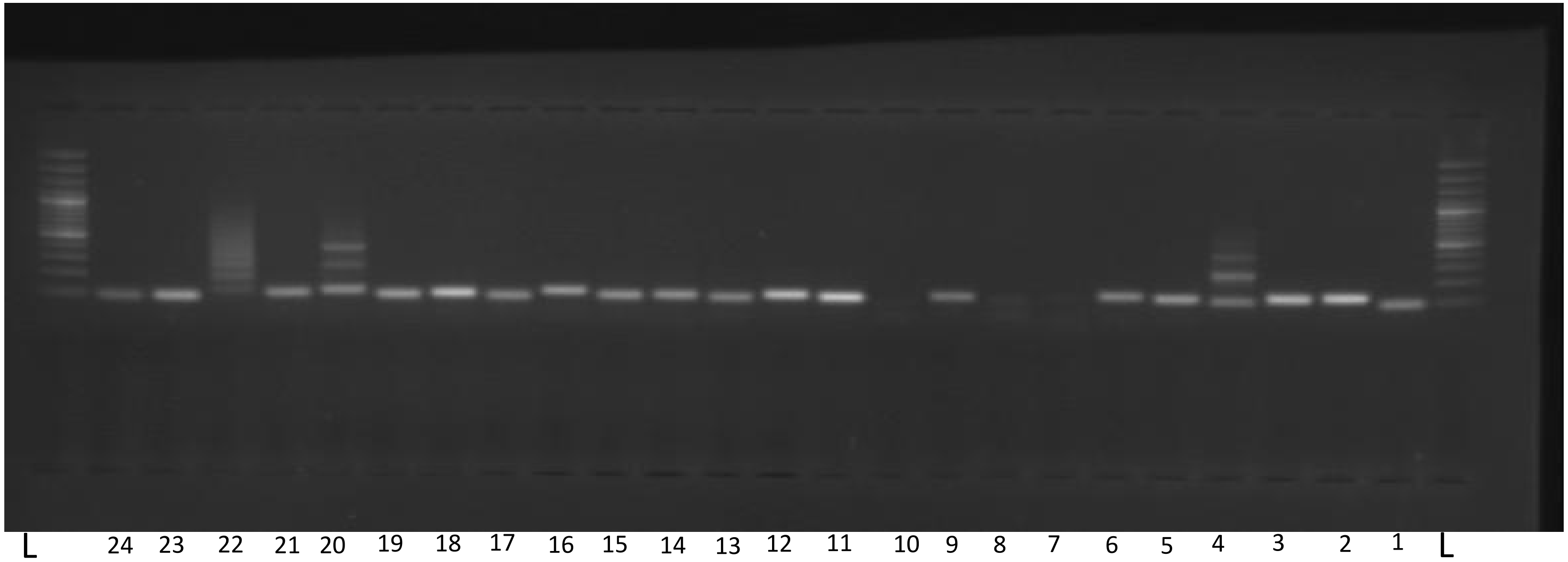

Supplement: Additional file 4 — Figure s4. Gel showing primer test to identified NB-LRRs in Norway spruce using primers designed from in silico studies of exiting spruce sequences in sequence databases. Only primer pairs giving a single band was further used for real-time RT-PCR. Therefore, NB-LRRs 22, 20, 10, 8 and 4 were not studied by qRT-PCR. [file 1471-2229-12-105-S4.pdf]
